# Language interventions for autistic people: An online survey of community member views and priorities

**DOI:** 10.1002/jcv2.70125

**Published:** 2026-04-11

**Authors:** Audrey Linden, Courtenay Norbury, Molly McCabe, Jo Saul, Estelle Killick, Evelyn Sam, Simone Dufresne, Freya Elise, Kurinchi Gurusamy, Laura Crane

**Affiliations:** ^1^ Centre for Research in Autism and Education (CRAE) UCL Institute of Education University College London London UK; ^2^ School of Psychology and Counselling Open University Milton Keynes UK; ^3^ Department of Clinical Educational and Health Psychology University College London London UK; ^4^ Eliot‐Pearson Department of Child Study & Human Development Tufts University Medford Massachusetts USA; ^5^ Department of Surgical Biotechnology University College London London UK; ^6^ Autism Centre for Education and Research (ACER), Department of Disability, Inclusion and Special Needs School of Education Birmingham UK

**Keywords:** autism, community involvement, ethics, interventions, language

## Abstract

**Background:**

Identifying interventions for developing language skills in autistic people is a top research priority. To develop effective language interventions, it is essential to understand whether community members feel they are important, acceptable, and meaningful. The aim of our research was to elicit views from members of the autism community on language and language interventions for autistic people.

**Methods:**

Our diverse team (comprising those with lived and professional experience of autism and/or language interventions) achieved our goals using an online survey. Via opportunity sampling, we recruited 356 participants including autistic adults, parents of autistic children, and professionals/researchers working with autistic people. Data were thematically analyzed.

**Results:**

Participants recognised the importance of language for self‐advocacy, societal participation, interacting with others, and expressing needs, feelings and desires. Language was seen as a potential tool for communication, but one of crucial importance in navigating a world designed for non‐autistic people. Responses also strongly emphasised the importance of language interventions being focused on autistic people's individual needs, that benefit them, allow them to be their authentic selves, and prioritise choice and agency.

**Conclusion:**

Our research highlights the importance of including community voices in the development of language interventions. Future language interventions should be individualised to a person's needs and wishes, respect autistic people's identity, and support self‐advocacy. Subsequent work should ensure that seldom‐heard voices are centered in such discussions.

## Key Points

**What's known?**

- Identifying interventions that support the language skills of autistic people is a top research priority. In addition to developing a strong evidence‐base, it is essential to determine community views on such interventions.

**What's new?**

- We examined the perspectives of members of the autism community on language and language interventions for autistic people. Our sample emphasised the importance of language for autistic people, and felt that interventions to support language needed to be neurodiversity‐affirming in their approach.

**What's relevant?**

- In designing new interventions to support the language skills of autistic people, or when evaluating existing interventions, it is essential for researchers and practitioners to reflect on the purpose of the intervention and the extent to which it aligns with the views and priorities of autism community members.

## INTRODUCTION

The heterogeneity of language profiles—within and across autistic people—has long been recognised (Boucher, 2012). While approximately 25% of autistic people speak few or no words (Rose et al., 2016; Tager‐Flusberg & Kasari, 2013), approximately 5% of autistic children show language trajectories like their non‐autistic peers (Pickles et al., 2014). Among autistic adults who use language, communication can fluctuate (Cummins et al., 2020) and can be an area of strength and/or interest (e.g., Ferreira et al., 2024; Kapp & Gudknecht, 2025). Given this variability, language is not part of diagnostic criteria for autism, but specification of accompanying language impairment is a component of clinical specifiers of severity (American Psychiatric Association, 2013) and sub‐categories (autism with/without language impairment; World Health Organisation, 2022).

There is debate around the causes of language differences experienced by some autistic people. Some theories center on differences in social cognition and social engagement (Williams et al., 2008), suggesting that interventions addressing social interaction and social cognitive processing (e.g., interventions targeting theory of mind skills, see Fletcher‐Watson et al., 2014) might have downstream effects on language development. In contrast, Kjelgaard and Tager‐Flusberg (2001) have argued that, at least for some autistic children, language difficulties may represent a co‐occurring condition. This perspective suggests that interventions should target language directly, providing autistic children with more structured exposure and practice with new language forms, ideally in functional contexts. Interventions that address language outcomes therefore vary considerably in their type, approach, and conceptual underpinnings (see Sandbank, Bottema‐Beutel, Crowley, Cassidy, Feldman, et al., 2020, for examples).

Identifying interventions that effectively support language skills in autistic people is a top research priority (Autistica, 2016). A meta‐analysis by Sandbank, Bottema‐Beutel, Crowley, Cassidy, Feldman, et al. (2020) examined the effects of a range of interventions focusing on language outcomes in autistic children, including behavioral interventions (e.g., the Picture Exchange Communication System; Bondy & Frost, 2001), developmental interventions (e.g., DIR/Floortime; Greenspan & Wieder, 2007), Natural Developmental Behavioral Interventions (e.g., the Early Start Denver Model; Rogers & Dawson, 2007), and sensory interventions (e.g., Sensory Integration Therapy; Ayres, 1972). These interventions include those aimed at targeting language directly, as well as those with likely downstream benefits on language development. Sandbank, Bottema‐Beutel, Crowley, Cassidy, Feldman, et al. (2020) reported significant, small‐to‐moderate improvements in language outcomes following intervention; and effects were more pronounced for expressive and composite, versus receptive, language. Stronger language skills at the start of the intervention were associated with greater language gains, and interventions led by clinicians (or clinicians working with caregivers) yielded larger effects than interventions delivered by caregivers alone. However, the authors highlighted the need for caution in interpreting their findings, given the need for improvements in study quality (cf. Bottema‐Beutel et al., 2023; Sandbank, Bottema‐Beutel, Crowley, Cassidy, Dunham, et al., 2020).

Alongside a pressing need for high‐quality research evidence on the effectiveness of interventions, there is growing recognition that interventions must be acceptable to the communities they seek to serve (Skivington et al., 2021). This imperative has been starkly illustrated in the field of autism, where systematic reviews and meta‐analyses have suggested strong evidence for some behavioral interventions, yet community consultation has identified evidence of unintended harm (see Leaf et al., 2022, for discussion). Community views may be particularly equivocal with respect to language intervention. In a recent study, Baiden et al. (2025) elicited the views of autistic adults on the use of common intervention goals and practices. While their study focused on behavioral interventions generally, their work highlighted an important paradox with respect to language interventions more specifically. In particular, their results demonstrated that promoting communication by focusing primarily on verbal speech was positively endorsed by just 13% of participants. However, verbal speech is arguably an important mechanism through which to achieve what participants described as “uncontroversial” goals such as reducing danger (endorsed by 96% of participants) and increasing independence (endorsed by 88% of participants).

The ethical imperative for autistic voices to be centered (as per Baiden et al., 2025) has been well documented (see, e.g., Milton et al., 2014). Yet complementary views from the wider community are also key, not least because parents/carers often consent to interventions on behalf of their autistic children, and as interventions are typically implemented and evaluated by clinicians and researchers. With respect to language interventions, it can also be challenging to directly access the perspectives of some members of the autistic community (e.g., those who do not have access to language). As such, members of the wider autism community (e.g., parents/carers and professionals) may be able to offer useful insights based on their experiences of supporting such individuals (e.g., Hersh et al., 2024).

In summary, while existing studies have examined community member views on autism research or interventions generally (Baiden et al., 2025; Hersh et al., 2024; Roche et al., 2021; Schuck et al., 2024), our study represents the first attempt to apply these efforts to language interventions specifically. We sought to gather the views of autistic people, parents/carers of autistic people and professionals (including researchers). Our specific research question was: what are the views and experiences of members of these groups with respect to language interventions? Our goal was to use the data gathered to inform future research and practice.

## METHODS

### Participatory methods

Community involvement strengthens the “rigor, relevance and reach of science” (Balazs & Morello‐Frosch, 2013, p. 10). Our diverse team included autistic and non‐autistic researchers, some of whom had additional connections to the autistic community (e.g., as professionals supporting autistic people, or as parents of autistic children). These team members were involved at every stage of the project, including: applying for funding; designing and conducting the research; and analysing, writing, and disseminating the findings (cf. Fletcher‐Watson et al., 2019). We also consulted with two community members recruited through the UK charity Autistica. Our consultants were an autistic adult who had previously received language intervention, and the parent of an autistic teenager who previously received language intervention. Our consultants provided input on survey design and development, including advising on survey questions and how best to make the survey accessible. Consultants were financially compensated for their time.

### Participants

Opportunity sampling was used to collect data from autistic adults, parents/carers of autistic people, and professionals (including researchers) working in the field (acknowledging participants' multiple intersecting identities). Our aim was to recruit participants with a range of experiences relating to language and language interventions, from across the UK (cf. Braun et al., 2021). To achieve this aim, we shared our research through various avenues, including charities, databases of research participants, social media, and personal contacts of the research team. Recruitment was open for 6‐weeks.

A total of 518 participants engaged with our survey. We removed 151 responses as they did not respond to questions around language/communication, and we removed a further 11 as they only responded to one of our two initial questions on language/communication. We therefore had 356 complete responses: 216 from autistic participants and 140 from non‐autistic participants (see Table 1 and Table S1). Our sample included 147 autistic adults with no other connection to autism, 140 parents/caregivers of autistic people (41 of whom were autistic), 100 professionals (42 of whom were autistic), and 35 researchers (18 of whom were autistic). Sixty‐eight percent of participants were female (with a particularly unrepresentative gender balance among our autistic sample, cf. Loomes et al., 2017), and 91% were white (which is slightly higher than representative, since census data suggests that around 80% of the UK population is white).

**TABLE 1:** Demographic characteristics of participants.

Group	Autistic (*n* = 216)	Non‐autistic (*n* = 140)
Autistic only	147	‐
Parent/carer	41	99
Professional	42	58
Researcher	18	17
Uses no spoken language	2	‐
Been through language intervention	37	‐
Parent of child who uses no spoken language	2	6
Parent of child who has been through language intervention	16	41

We acknowledge that the nature of our methods precluded the direct involvement of autistic individuals who did not have access to language and, within the context of this limitation, we were conscious of the need to include a diversity of perspectives. To that end, we noted that two autistic adults who did not use spoken language participated (as they were able to write their responses). We also captured the views of eight parents/carers of autistic children who did not use spoken language (two of whom were autistic themselves). Our sample also included 37 autistic people who had experienced a language intervention, and 57 parents/carers of an autistic person who had experienced a language intervention (16 of whom were autistic themselves). These participants—as well as the professionals involved in the delivery and evaluation of language interventions—may have been particularly well placed to comment on language interventions. To assess bias, we analyzed the make‐up of the excluded sample who had answered demographic questions. There were no systematic differences in those included and excluded from the survey (see Table S2).

### Materials

We designed our online survey (as per Braun et al., 2021) with reference to guidelines for accessible writing (Mencap, 2023) and guidelines for the inclusion of autistic participants (Nicolaidis et al., 2019). We also discussed accessibility challenges and solutions with our consultants. Accommodations implemented included: using simple and concrete language; providing subtitles to accompany videos; providing options to respond via multiple modes; and providing inclusive response options. The final survey (see Appendix S1) was hosted on Qualtrics, with participants able to respond in writing, or to upload audio, video, or visual‐based responses. Likewise, participants could complete the survey via phone or video call with a member of the research team, which one participant did. The survey was designed to take approximately 15 min to complete. No incentives were offered for completion.

The survey was introduced as research into community perceptions about language interventions for autistic people and was organised into five sections. Section One provided study information (via a short video, and written information), and the opportunity to provide informed consent. Section Two collected demographic information, including participants' connection to autism. Additional demographics were collected based on these connections (e.g., autistic people were asked for details of their diagnosis; parents/caregivers were asked for details of their child's diagnosis).

Section Three focused on language and communication. First, definitions of communication and language were provided, with the goal that participants approached subsequent questions with shared understanding. Participants rated how important (a) communication and (b) language were to them on a scale from 0 (not important) to 10 (very important), explaining their answer in an open text box. We included questions about language and communication separately to encourage participants to consider and differentiate between both constructs. Participants were asked whether they thought language was important for autistic people (yes/no/unsure) and to explain their answer in an open text box.

Section Four involved presenting participants with a short (3 min) video, with a transcript. This (optional) video provided a definition of what an intervention is along with brief examples of a range of language interventions (e.g., behavioral interventions, technology interventions) adapted from a prior review by Sandbank, Bottema‐Beutel, Crowley, Cassidy, Feldman, et al. (2020). Section Five asked: (1) What do you think are the benefits of language interventions for autistic people? (2) What do you think are the potential disadvantages/harms of language interventions for autistic people? The survey closed by thanking participants and offering them the opportunity to participate in focus groups. These focus groups were initially planned to gather more in‐depth data on the topics noted in the survey and to explore any areas of ambiguity. However, given the clarity, richness and depth of survey responses, our team decided (via reflexive discussion) that supplementary focus groups were unnecessary.

### Data analysis

Prior to analysis, we checked our data for indicators of fraudulent responses, such as bots or repeat responders, and no indicators suggested cause for concern (Johnson et al., 2024).

Responses to closed questions were analyzed descriptively using R. We analyzed data from autistic and non‐autistic participants separately, irrespective of other connections to autism (e.g., if a participant was autistic and worked professionally with autistic people, their responses were grouped with those of other autistic respondents).

Responses to open‐ended questions were analyzed using reflexive thematic analysis (Braun & Clarke, 2006, 2013, 2019, 2021) using an inductive (bottom‐up) approach to identify patterns within the dataset. We approached our analysis from a critical realist perspective: participants' accounts were taken as being true to them while also mediated by features of the broader social context. As such, multiple valid accounts regarding language and language interventions may be represented (Houston, 2001; Terry et al., 2017; Willig, 2013). Analysis was led by three members of the research team (autistic and non‐autistic researchers), with regular input from other team members (who supportively challenged the analytic process through peer discussion and written feedback on developing themes). Team members recursively proceeded through stages of (1) data familiarisation, (2) initial code generation, (3) searching for themes, (4) theme review, (5) naming and defining themes, and (6) report production. During data familiarisation and initial code generation, we considered responses from autistic participants followed by responses of non‐autistic participants. Due to considerable overlap between these groups, remaining analyses were completed for the dataset overall, while taking care to note any differences between groups (autistic vs. non‐autistic) and sub‐groups (e.g., parents, professionals, researchers).

To ensure the rigor and quality of our analyses, we closely attended to our positionality (cf. Braun & Clarke, 2019, 2021). All involved had personal and/or professional connections to autism, with varying—often contrasting—perspectives on language interventions for autistic people (e.g., around the relative importance of oral language as compared to other forms of communication). This diversity deepens confidence in the dependability and trustworthiness of qualitative results (Cascio et al., 2019). We engaged in reflexive discussions throughout the analytic and writing‐up process, and we conducted a negative case analysis (specifically searching for quotes and ideas that contradicted our identified themes and our personal views) to ensure we were not overlooking perspectives that differed to our own.

### Procedure

Ethical approval was obtained from the Research Ethics Committee at IOE, UCL's Faculty of Education and Society (REC 1664) on 27th July 2022. Our survey was open from 10th October to 14th November 2022.

## RESULTS

First, we analyzed numerical scores of the importance of (a) language and (b) communication for autistic people. As seen in Table 2 and Table S3, there was convergence between ratings provided by autistic and non‐autistic respondents. Mean scores suggested that both groups rated communication more highly than language, and this was consistent across all subgroups (i.e., whether respondents were also parents, professionals and/or researchers).

**TABLE 2:** Ratings of the importance of language and communication.

	Importance of language (0–10)	Importance of communication (0–10)
	Mean (SD)	Mean (SD)
Autistic (*n* = 216)	8.3 (2.3)	9.1 (1.8)
Non‐autistic (*n* = 140)	8.0 (1.9)	9.6 (1.0)

We identified three themes from the qualitative data, evident across the sample: (1) *Language is just one possible tool for communication*; (2) “*Autistic people don't live on autism island*”; and (3) *We must put autistic people first*. Illustrative quotes for each theme are provided, and participant numbers are accompanied by indicators of whether participants are autistic (a) or non‐autistic (*n*).

### Theme 1: Language is just one possible tool for communication

Our participants highlighted how language was important for autistic people's basic needs and functioning, physical and mental wellbeing, connection with others, and to facilitate fulfilment of potential: “it is a tool, one which can be used in a number of different ways, starting at ground level, personal safety, up to delivering a thesis on their art or skill” (a, 102). Participants emphasised the importance of autistic people being able to express their needs and feelings and forming and maintaining relationships with others; with verbal language perceived as facilitating such outcomes and, in turn, reducing isolation and improving wellbeing. Some participants related verbal language as key to their own or their autistic child's autonomy and independence (e.g., being understood in school and employment).

Yet participants emphasised that language was only one aspect of communication. Communication was viewed as a two‐way endeavor: the onus for successful communication should not solely lie with autistic people adjusting to meet non‐autistic norms. Instead, participants felt that non‐autistic people had a part to play in understanding autistic people and responding to their ways of communicating. As one autistic participant (76) commented: “[non‐autistic] people require interventions to help autistic people. For example, using language in a precise manner, rather than expecting an autistic person to ‘read between the lines’”.

Language and communication were not always differentiated by participants, with many using the terms interchangeably. However, there were clear statements around oral language. Specifically, some participants emphasised that—for those who had a choice—oral language was not always a preference for all autistic people. Even those who usually communicated verbally felt that oral language was not always accessible to them: “I get very frustrated when my words don't work, which tends to happen if I am stressed and/or very tired” (a, 112). For others, oral language was a particular strength, interest or passion: “I love the English language ‐ how it sounds, how words and phrases are constructed. I enjoy finding the exactly appropriate word or phrase for what I'm writing” (a, 386). Participants emphasised the need for an individualised approach to language.

In terms of language interventions, participants noted that these needed to be respectful to autistic ways of communicating. For example, participants emphasised the importance of allowing autistic people to remain their authentic selves, tailoring interventions according to autistic people's needs and preferences, and ensuring participants retained choice and agency. A crucial consideration for any intervention was avoidance of harm. As one autistic participant, who was also a parent (42) noted: “so much of the world is built on language, it would make life difficult if you didn't use it…. [but] teaching autistics to communicate like [non‐autistic people] can be harmful due to teaching masking and giving the idea that [non‐autistic] communication is superior.”

In summary, the key message from this theme was one of language being a possible tool for communicating, which tended to be highly valued. Yet participants also emphasised how individual differences in language use needed to be considered. For example, access to language could fluctuate depending on the situation, and using oral language may not be a person's preference.

### Theme 2: “Autistic people don't live on autism island”

Related to the importance of language as one possible tool for communication, participants explicitly commented that language was necessary for autistic people to communicate with others and get by in the world. As one autistic participant (106, also a parent and a professional) explained: “autistic people don't live on “autism island”, we live in a world that is made by and for neurotypical people. To live well here, it's much easier if we have language”. Participants further commented on ethical issues around ensuring that language is not denied to autistic people as they could be seriously disadvantaged and/or disabled without it. Such concerns ranged from autistic people needing language to be able to access their basic needs, to needing language to be able to thrive in different areas of their lives. Some participants commented that language could allow non‐autistic people insight into autistic people's worlds, perhaps aiding understanding of autistic people's experiences.

Participants noted that reliance on language could place the burden of responsibility onto autistic people to accommodate their communication to that of the non‐autistic majority: “we won't make progress on our own rights if we can't discuss them with people who don't understand them” (a, 385). Indeed, some participants emphasised the need to reduce this imbalance, with non‐autistic people needing to make more effort: “The burden of improving communication should be shared ‐ but [non‐autistic] people are often either unwilling to make allowance, or ignorant of the need to do so” (a, 392). Participants emphasised the need to improve understanding among non‐autistic people, whereas it was perceived that existing interventions concentrated on the autistic person alone.

In summary, the key message from this theme was that language was highly valued and can increase opportunity. Nevertheless, there was frustration that non‐autistic people do not put enough effort into understanding and accommodating differences in autistic communication. Greater acceptance and willingness to adapt to the needs of autistic partners could make communication easier and more successful, whatever the individual's level of language.

### Theme 3: We must put autistic people first

Participants emphasised the variability in language interventions and how these were utilised. As one participant (*n*, 495, parent, professional and researcher) explained:

[if someone has] perfect language but needed help with nuances and understanding metaphor etc… would this sort of help constitute ‘language intervention’?

[if someone] uses spoken language but finds things like pronouns a bit tricky ‐ for example, using ‘you’ to mean ‘one’ as opposed to ‘you’ second person singular ‐ teaching him about this and other grammatical quirks: is that language intervention?

Then you have a very classic [behavioral intervention] ‐ for some teaching immediate skills so that they are not frustrated by not being able to communicate their need can be extremely beneficial ‐ but, there may be other ways.

For some [picture communication systems] might work best, again because their own gestures might not be universally understood ‐ but some have argued it can delay language ‐ is this intervention useful in the long term?”

Yet irrespective of the type of language intervention, participants expressed a desire to put autistic people first. Four sub‐themes were identified: (1) interventions being for autistic people's benefit and in line with their goals; (2) interventions allowing autistic people to retain their authentic sense of self; (3) tailoring interventions to individual needs and preferences; and (4) retaining choice and agency in interventions. There was also a minority of participants—from across the sample—who stated that they did not see any potential risk of harm from participants taking part in language interventions.

#### Sub‐theme 1: Interventions for the benefit of autistic people and in line with their goals

Participants emphasised prioritising the needs and wishes of autistic people who are offered interventions; ensuring the intervention benefits them and is aligned with their goals. Some participants referred to the benefits of being supported to communicate verbally, and opportunities that would become more accessible as a result. However, participants also mentioned the need to consider whose goals are at the center of the intervention, emphasising the focus needed to be on the autistic person. As one non‐autistic participant (151, a parent and professional) noted: “language interventions can enhance a child's confidence and help them find their unique place in the world. An intervention should empower a child, that is, allow them to refuse and express preferences.”

#### Sub‐theme 2: Retaining autistic sense of self

Participants expressed concern regarding the goals of some interventions, emphasising how autistic people should not be pushed through interventions to appear less autistic, and that their strengths and identity must be kept intact. Potential harms of interventions (e.g., in situating autistic people as wrong or as being the problem), were mentioned: “teaching us to mimic neurotypical speech and behavior can be devastating to our sense of self and to self‐acceptance and feeling we are OK as autistic people” (a, 330). As one participant emphasised, we need to move away from interventions “focused on changing the autistic person, rather than on helping those who love them, work with them and, ultimately, communicate with them, understand them better” (*n*, 495, parent, professional and researcher).

#### Sub‐theme 3: Tailoring interventions to autistic people's needs and preferences

Participants commented on the need to tailor interventions to individuals' needs. Participants wanted interventions targeting areas that an individual may be struggling with, while avoiding blanket approaches that could be mistargeted and “very condescending” (a, 354). Since language was not perceived as accessible to, or preferred by, all autistic people, participants stressed that interventions needed to prioritise the unique needs of each autistic person, irrespective of their preferred methods of communicating: “there are many different ways now that people with autism can communicate without using language. It gives them a range of interventions which will suit individuals” (*n*, 482, parent).

#### Sub‐theme 4: Retaining choice and agency in interventions

Participants discussed the importance of ensuring that intervention recipients (especially children), are given choice over participating and are supported to advocate for themselves. One non‐autistic participant suggested that: “one of the first lessons [of a language intervention] might need to be how to say no to lessons” (*n*, 2, professional and researcher). Ethical considerations were highlighted, including that interventions should not be forced on participants, and autistic people must be listened to and heard.

In summary, the key message across these sub‐themes was around putting autistic people first. Participants had differing, and occasionally uncertain, views around what constituted a language intervention. However, irrespective of the specific approach adopted, it was felt to be important to tailor interventions to the individual, to ensure interventions are focused on their needs, and to afford individual choice and agency. Crucially, it was not deemed appropriate for interventions to seek to change an individual's autistic identity.

## DISCUSSION

Our research involved gathering, for the first time, perspectives from members of the autism community (including autistic people, parents/carers and professionals) on language and language interventions for autistic people. From our qualitative analysis, we identified three key themes: (1) Language is just one possible tool for communication; (2) “Autistic people don't live on autism island”; and (3) We must put autistic people first. To summarise, participants recognised the importance of language for self‐advocacy, societal participation, interacting with others, and for expressing needs, feelings, and desires. Language was seen as one possible tool for communication, but one of crucial importance in navigating a world designed for non‐autistic people. Respondents also emphasised the importance of language interventions focused on individual needs of autistic people, that benefitted them, that allowed them to be their authentic selves, and that prioritised choice and agency (cf. Leadbitter et al., 2021).

Before considering the findings in more depth, it should be noted that while some participants were discussing interventions specifically targeted at supporting language, others referred to broader autism‐focused interventions that had a language or communication component, and some participants referred to both (often interchangeably). This outcome potentially reflects the short video we presented to participants, which provided examples of language interventions grounded in different theoretical approaches. If participants align more with the notion that language and autism are separate (cf. Kjelgaard & Tager‐Flusberg, 2001; Loucas et al., 2008), interventions directly targeting language will be discussed. If, instead, participant relate language differences to autistic social differences (Williams et al., 2008), they will refer to broader interventions that have downstream impacts on language development. While the latter was not explicitly discounted by our sample, they did oppose any intervention aimed at normalising autistic individuals to enhance their language and communication skills; a finding supported by autistic community views on interventions more broadly (e.g., Hersh et al., 2024; Schuck et al., 2024).

Crucially, language interventions can be neuro‐affirming in approach. Indeed, our sample highlighted how a major goal of language interventions should be to ensure that autistic people have access to language skills as a possible tool for communication. Failure to offer language as a tool was felt to significantly limit autistic people's opportunities. Lack of language may also increase vulnerability to negative experiences, such as undiagnosed medical problems or abuse, because specific messages regarding past experiences or internal discomfort may not be communicated precisely. Indeed, interventions that aim to reduce danger and self‐injurious behavior have been shown to be highly valued by the autistic community (Baiden et al., 2025). Our participants therefore expressed a need for language being taught as one potential tool for communication, in a neuro‐affirming way.

Relatedly, choice and agency were strongly valued by our sample. Where autistic people are able, they should participate in decisions about what language targets are most meaningful to them and how interventions are delivered and monitored (Schuck et al., 2024). However, a potentially contentious issue is how best to proceed when an individual does not have foundational language skills to convey their choices and assert their agency; a sub‐sample not directly represented in our survey. In such cases, people with a caring role in their life may need to make decisions on their behalf, and this can be challenging when they may not know what is in the person's best interests, or where their interests may be competing or conflicting. In such cases, a cautious and nuanced approach is key, balancing the meaningful benefits of language interventions against the risk of potential harm. The input of autistic adults in setting intervention goals for autistic children has been proposed (see Schuck et al., 2024), along with a need for intervention studies to measure and report on harms (cf. Dawson & Fletcher‐Watson, 2022). Crucially, long‐term follow up in intervention studies is extremely rare, yet potential harms (e.g., masking) may not become evident until sometime later, and intervention impacts could be cumulative (Raymaker et al., 2020).

### Limitations

As with many online survey studies, we faced issues of representativeness (cf. Rubenstein & Furnier, 2021). First, autistic women were over‐represented in the sample relative to population norms. Second, we acknowledge that there was little ethnic diversity in our sample. While our sample broadly reflects the ethnic majority in the UK (where around 80% of the population are white), we acknowledge that some potential views and priorities (e.g., relating to the cultural responsiveness of interventions) are not captured. Finally, we acknowledge that our survey was only accessible to autistic people who have access to spoken or written language. As such, the first‐hand perspectives of those who have little or no access to language at all (around 25% of autistic people) are only indirectly represented, via the experiences of parents/carers and professionals. Often, directly incorporating the views and experiences of this under‐studied group requires a radically different approach (see Lewis‐Dagnall et al., 2023, for examples). Until then, it will be essential to exert caution in generalising the findings from this study to all autistic people.

## CONCLUSIONS

Our research represents the first community consultation focused on language interventions for autistic people. We highlight the importance of including members of the autism community in decisions about language interventions, to complement evidence around ‘what works’. We amplify their plea that future language interventions are designed and/or adapted with community input, individualised to people's needs, and respect autistic identities and experiences. In developing language interventions, it is essential that they serve autistic individuals by giving them vital tools for self‐advocacy and opportunity to engage with the wider world to the extent they wish to. Any interventions should be rigorously evaluated, both to establish the efficacy of the intervention, but also how important, acceptable, and meaningful the intervention is to the community it seeks to serve.

## AUTHOR CONTRIBUTIONS

**Audrey Linden**: Conceptualization; investigation; funding acquisition; writing—original draft; methodology; writing—review and editing; formal analysis; data curation. **Courtenay Norbury**: Conceptualization; investigation; funding acquisition; writing—review and editing; methodology; supervision; formal analysis. **Molly McCabe**: Writing—review and editing; methodology; data curation; formal analysis. **Jo Saul**: Writing—review and editing; supervision; formal analysis. **Estelle Killick**: Methodology; writing—review and editing; formal analysis; data curation. **Evelyn Sam**: Writing—review and editing; methodology; formal analysis; data curation. **Simone Dufresne.** Methodology; investigation; writing—review and editing; formal analysis; data curation. **Freya Elise**: Conceptualization; investigation; funding acquisition; methodology; writing—review and editing; data curation; formal analysis; **Kurinchi Gurusamy**: Conceptualization; funding acquisition; methodology; writing—review and editing; supervision; **Laura Crane**: Conceptualization; investigation; funding acquisition; writing—original draft; methodology; writing—review and editing; formal analysis; project administration; data curation; supervision.

## CONFLICT OF INTEREST STATEMENT

Professor C.N. is President of NAPLIC—an organisation for professionals who support young people's language in school settings. She does not receive any financial numeration for this role. The remaining authors have declared that they have no competing or potential conflicts of interest.

## ETHICAL CONSIDERATIONS

Informed consent was obtained from all participants. Ethical approval was granted via the IOE—UCL's Faculty of Education and Society Research Ethics Committee on 27 July 2022 (ref: REC1664).

## Supporting information

Supporting Information S1

## Data Availability

Research data are not shared.
