# Supplementary material for: Language interventions for autistic people: An online survey of community member views and priorities
Source: JCPP Adv. 2026 Apr 11:e70125. Online ahead of print. doi: 10.1002/jcv2.70125 (PMC13338969; doi:10.1002/jcv2.70125)
Supplement: Supplementary file 1 — Supporting Information S1 [file JCV2-9999-e70125-s001.docx]

**Language interventions for autistic people: an online survey of community member views and priorities**

**Supporting Information**

Table S1. Demographic characteristics of participants

| Demographic |  | Autistic (n=216) | Non-autistic (n=140) |
| --- | --- | --- | --- |
| Capacity in which survey was completed | Autistic only | 147 (68.1%) | 0 |
|  | Family member | 41 (19.0%) | 99 (70.7%) |
|  | Professional | 42 (10.4%) | 58 (41.4%) |
|  | Researcher | 18 (8.3%) | 17 (12.1%) |
| Gender identity | Man (including trans men) | 58 (26.9%) | 22 (15.7%) |
|  | Woman (including trans women) | 127 (58.8%) | 114 (81.4%) |
|  | Non-binary or gender nonconforming | 20 (9.2%) | 2 (1.4%) |
|  | Other | 11 (5.1%) | 0 |
|  | Not stated | 0 | 2 (1.4%) |
| Age | Mean (SD) | 43.82 (14.95) | 45.76 (10.35) |
| Ethnicity | White/White British | 198 (91.7%) | 125 (89.3%) |
|  | Asian/Asian British | 2 (0.9%) | 2 (1.4%) |
|  | Black/African/Caribbean/Black British | 2 (0.9%) | 1 (0.7%) |
|  | Mixed/Multiple ethnic groups | 3 (1.4%) | 2 (1.4%) |
|  | Other ethnic group | 8 (3.7%) | 6 (4.3%) |
|  | Prefer not to say | 3 (1.4%) | 2 (1.4%) |
| Diagnosis of autism | Formal diagnosis | 195 (90.3%) |  |
|  | Self-identify | 18 (8.3%) |  |
|  | Prefer not to say | 3 (1.4%) |  |
| Age of diagnosis | Mean (SD) | 35.83 (16.32) |  |
| Uses spoken language | Yes | 213 (98.6%) |  |
|  | No | 2 (0.9%) |  |
|  | Prefer not to say | 0 |  |
| Uses alternative forms of communication (PECS, sign language, AAC) | Yes | 17 (7.9%) |  |
|  | No | 197 (91.2%) |  |
|  | Prefer not to say | 1 (0.5%) |  |
| Co-occurring intellectual/learning disability | Yes | 30 (13.9%) |  |
|  | No | 182 (84.3%) |  |
|  | Prefer not to say | 2 (0.9%) |  |
| Additional diagnosis | ADHD | 43 (19.9%) |  |
|  | Language disorder | 2 (0.9%) |  |
|  | Literacy disorder (e.g., dyslexia) | 22 (10.2%) |  |
|  | Genetic chromosomal condition (e.g., Down Syndrome) | 0 |  |
|  | Mental health condition (e.g., anxiety, depression) | 133 (61.6%) |  |
|  | Tourettes | 2 (0.9%) |  |
|  | Other diagnosis | 56 (25.9%) |  |
|  | Prefer not to say | 1 (0.5%) |  |
| Formal assessment by SLT | Yes | 40 (18.5%) |  |
|  | No | 172 (79.6%) |  |
|  | Prefer not to say | 2 (0.9%) |  |
| Been through language intervention | Yes | 37 (17.1%) |  |
|  | No | 176 (81.5%) |  |
|  | Prefer not to say | 2 (0.9%) |  |
| Parent and caregivers  (Note that percentages presented below are percentages within the category of parents/children) |  | 41 (19.0%) | 99 (70.7%) |
| Child’s age | Mean age in years (SD) | 19.03(9.62) | 15.44(7.79) |
| Child’s gender identity | Male; including trans male | 22 (53.7%) | 68 (68.7%) |
|  | Female; including trans female | 14 (34.1%) | 27 (27.3%) |
|  | Non-binary or gender nonconforming | 3 (7.3%) | 3 (3.0%) |
|  | Other (please specify) | 2 (4.9%) | 1 (1.0%) |
| Child’s formal diagnosis | Yes | 34 (82.9%) | 96 (97.0%) |
|  | No | 7 (17.1%) | 3 (3.0%) |
|  | Prefer not to say | 0 | 0 |
| Child’s age of diagnosis | Mean age in years (SD) | 11.6(10.2) | 5.5(3.5) |
| Child uses spoken language | Yes | 39 (95.1%) | 93 (93.9%) |
|  | No | 2 (4.9%) | 6 (6.1%) |
|  | Prefer not to say | 0 | 0 |
| Child uses alternative forms of communication (PECS, sign language, AAC)? | Yes | 9 (22.0%) | 26 (26.3%) |
|  | No | 32 (78.0%) | 73 (73.7%) |
|  | Prefer not to say | 0 | 0 |
| Child have a co-occurring intellectual/learning disability | Yes | 13 (31.7%) | 44 (44.4%) |
|  | No | 28 (62.3%) | 52 (52.5%) |
|  | Prefer not to say | 0 | 1 (1.0%) |
|  | No response given | 0 | 2 (2.1%) |
| Child have any additional diagnoses | ADHD | 6 (14.6%) | 11 (11.1%) |
|  | Language disorder | 2 (4.9%) | 4 (4.0%) |
|  | Literacy disorder (e.g., dyslexia) | 2 (4.9%) | 5 (5.1%) |
|  | Genetic chromosomal conditions (e.g., Down Syndrome) | 0 | 2 (2.0%) |
|  | Mental health condition (e.g., anxiety, depression) | 6 (14.6%) | 22 (22.2%) |
|  | Tourette's | 1 (2.4%) | 1 (1.0%) |
|  | Other diagnosis | 13 (31.7%) | 28 (28.3%) |
|  | Prefer not to say | 0 | 3 (3.0%) |
| Child been formally assessed by a SLT | Yes | 22 (53.7%) | 76 (76.8%) |
|  | No | 18 (43.9%) | 21 (21.2%) |
|  | Unsure | 1 (2.4%) | 2 (2.0%) |
|  | Prefer not to say | 0 | 0 |
| Child gone through a language intervention | Yes | 16 (39.0%) | 41 (41.4%) |
|  | No | 25 (61.0%) | 48 (48.5%) |
|  | Unsure | 0 | 10 (10.1%) |
|  | Prefer not to say | 0 | 0 |
| Professionals  (note that percentages below represent percentage of professionals who are autistic/non-autistic) |  | 42 (19.4%) | 58 (41.4%) |
| Professional role | Speech-language therapist/assistant | 3 (7.1%) | 14 (24.1%) |
|  | Teacher/teaching assistant | 14 (33.3%) | 19 (32.8%) |
|  | Support worker | 7 (16.7%) | 7 (12.1%) |
|  | Medical Professional (MD, RN, GP, etc) | 1 (2.4%) | 1 (1.7%) |
|  | Medical Professional (Mental health practitioner) | 1 (2.4%) | 3 (5.2%) |
|  | Other (for example SENCO, peer researcher, SEND adviser). Note most who stated ‘other’ did not enter a text response. | 16 (38.1%) | 14 (24.1%) |
|  | Prefer not to say | 0 | 0 |
| Years of experience working with autistic people | 0 - 4 years | 7 (16.7%) | 10 (17.2%) |
|  | 5 - 10 years | 12 (28.6%) | 16 (27.6%) |
|  | 10+ years | 19 (45.2%) | 29 (50.0%) |
|  | Prefer not to say | 0 | 0 |
|  | Did not respond | 4 (9.5%) | 3 (5.2%) |
| Know someone who has gone through a language intervention | Yes | 6 (14.3%) | 6 (10.3%) |
|  | No | 27 (64.3%) | 42 (72.4%) |
|  | Unsure | 5 (11.9%) | 6 (10.3%) |
|  | Prefer not to say | 0 | 1 (1.7%) |
|  | Did not respond | 4 (9.5%) | 3 (5.2%) |
| Deliver language interventions | Yes | 26 (61.9%) | 34 (58.6%) |
|  | No | 7 (16.7%) | 15 (25.9%) |
|  | Unsure | 5 (11.9%) | 6 (10.3%) |
|  | Prefer not to say | 0 | 0 |
|  | Did not respond | 4 (9.5%) | 3 (5.2%) |
| Researchers |  | 18 (8.3%) | 17 (12.1%) |
| Research role | Graduate student Masters level | 7 (38.9%) | 4 (23.5%) |
|  | Graduate student PhD level | 3 (16.7%) | 6 (35.3%) |
|  | Academic (early career) | 2 (11.1%) | 3 (17.6%) |
|  | Academic (mid-career) | 4 (22.2%) | 4 (23.5%) |
|  | Other (Please specify) | 2 (11.1%) | 0 |
|  | Academic (late career) | 0 | 0 |
| Years of experience working with autistic people | 0 - 4 years | 11 (61.1%) | 10 (58.8%) |
|  | 5 - 10 years | 3 (16.7%) | 3 (17.6%) |
|  | 10+ years | 3 (16.7%) | 4 (23.5%) |
|  | Prefer not to say | 1 (5.5%) | 0 |
| Know someone who has gone through a language intervention | Yes | 4 (22.2%) | 3 (17.6%) |
|  | No | 11 (61.1%) | 10 (58.8%) |
|  | Unsure | 3 (16.7%) | 4 (23.5%) |
|  | Prefer not to say | 0 | 0 |
| Conduct language interventions | Yes | 16 (88.9%) | 14 (82.4%) |
|  | No | 2 (11.1%) | 3 (17.6%) |
|  | Unsure | 0 | 0 |
|  | Prefer not to say | 0 | 0 |

Table S2. Key demographic characteristics of excluded participants

| Demographic |  | Autistic (n=50) | Non-autistic (n=20) |
| --- | --- | --- | --- |
| Capacity in which survey was completed (n=70 answered) | Autistic only | 32 |  |
|  | Family member | 13 | 11 |
|  | Professional | 7 | 10 |
|  | Researcher | 3 | 3 |
| Uses spoken language (n=45 answered) | Yes | 45 |  |
|  | No | 0 |  |
|  | Prefer not to say | 0 |  |
| Been through language intervention (n=43 answered) | Yes | 10 |  |
|  | No | 33 |  |
|  | Prefer not to say | 0 |  |
| Parent and caregivers |  | 13 | 11 |
| Child uses spoken language (n=20 answered) | Yes | 9 | 10 |
|  | No | 0 | 1 |
|  | Prefer not to say | 0 | 0 |
| Child gone through a language intervention (n=20 answered) | Yes | 4 | 4 |
|  | No | 4 | 4 |
|  | Unsure | 1 | 3 |

Note that there were 162 excluded participants. Demographics are provided for those who answered the selected question.

Table S3. Ratings of the importance of language and communication

|  | Importance of language  (0-10) | Importance of communication (0-10) |
| --- | --- | --- |
|  | *Mean (SD)* | *Mean (SD)* |
| Autistic overall (i.e., all groups, n = 216) | 8.3 (2.3) | 9.1 (1.8) |
| Autistic only | 8.4 (2.2) | 8.8 (2.1) |
| Autistic + parents | 8.5 (2.0) | 9.5 (1.0) |
| Autistic + professional | 7.6 (1.9) | 9.6 (0.8) |
| Autistic + researcher | 7.8 (2.3) | 9.5 (1.0) |
| Autistic + parents + professional | 7.8 (3.7) | 9.7 (1.0) |
| Autistic + parents + researcher | 8.3 (1.2) | 10.0 (0) |
| Autistic + parents + professional + researcher | 8.4 (2.3) | 10.0 (0) |
| Non-autistic overall (i.e., all groups, n = 140) | 8.0 (1.9) | 9.6 (1.0) |
| Non-autistic + parents | 8.3 (1.8) | 9.5 (1.2) |
| Non-autistic + professional | 7.5 (2.1) | 9.8 (1.0) |
| Non-autistic + researcher | 7.5 (2.2) | 10.0 (0) |
| Non-autistic + parents + professional | 8.0 (1.8) | 9.9 (0.3) |
| Non-autistic + parents + researcher | 10.0 (0) | 10 (0) |
| Non-autistic + parents + professional + researcher | 8.3 (1.2) | 10.0 (0) |

Appendix S1. Online Survey

**Demographics**

Demographics: This section has 4 questions.

What is your gender identity?

- Male; including trans male
- Female; including trans female
- Non-binary or gender nonconforming
- Other (please specify) [open text box]

What is your age? [open text box]

What is your ethnicity?

- White/White British Qualtrics
- Asian/Asian British
- Black/African/Caribbean/Black British
- Mixed/Multiple ethnic groups
- Other ethnic group [open text box]
- Prefer not to say

Do you use English as an additional language?

- Yes
- No
- Prefer not to say

What is your connection to autistic people? (check all that apply)

- I am an autistic person
- I am a parent or parental caregiver of an autistic person
- I am a professional who supports autistic people
- I am a researcher in the field of autism research

**Autistic Person Demographics**

Autistic Person Demographics: This section has up to 12 questions.

Has a professional diagnosed you as autistic? (if you self-identify as autistic you can still take part in the survey)

- Yes
- No
- Prefer not to say

Do you self identify as an autistic person?

- Yes
- No
- Prefer not to say

What was your age at diagnosis? [open text box]

Do you use spoken language?

- Yes
- No
- Prefer not to say

Do you use alternative forms of communication (PECS, sign language, AAC)?

- Yes
- No
- Prefer not to say

Do you have a co-occurring intellectual/learning disability?

- Yes
- No
- Prefer not to say

Do you have any of the following additional diagnoses?

- ADHD
- Language disorder
- Literacy disorder (e.g., dyslexia)
- Genetic chromosomal condition (e.g., Down Syndrome)
- Mental health condition (e.g., anxiety, depression)
- Tourettes
- Other diagnosis [open text box]
- Prefer not to say

Have you been formally assessed by a speech and language therapist?

- Yes
- No
- Prefer not to say

At any point in your life, have you gone through a language intervention? *Interventions are actions or treatments to try to improve something.*

- Yes
- No
- Prefer not to say

What types of language interventions have you gone through? [open text box]

**Caregiver Demographics**

Caregiver Demographics: This section has up to 12 questions.

What is your child's age? [open text box]

What is your child’s gender identity?

- Male (including trans male)
- Female (including trans female)
- Non-binary
- Prefer not to say

Does your child have a formal diagnosis of autism spectrum disorder?

- Yes
- No
- Prefer not to say

What was your child's age at diagnosis? [open text box]

Does your child use spoken language?

- Yes
- No
- Prefer not to say

Does your child use alternative forms of communication (PECS, sign language, AAC)?

- Yes
- No
- Prefer not to say

Does your child have a co-occurring intellectual/learning disability?

- Yes
- No
- Prefer not to say

Does your child have any of the following additional diagnoses?

- ADHD
- Language disorder
- Literacy disorder (e.g., dyslexia)
- Genetic chromosomal condition (e.g., Down Syndrome)
- Mental health condition (e.g., anxiety, depression)
- Tourettes
- Other diagnosis [open text box]
- Prefer not to say

Has your child been formally assessed by a speech and language therapist?

- Yes
- No
- Unsure
- Prefer not to say

Has your child gone through a language intervention?

- Yes
- No
- Unsure
- Prefer not to say

What types of language interventions has your child gone through? [open text box]

**Professional Demographics**

Professional Demographics: This section has up to 5 questions.

What is your professional role?

- Speech language therapist/assistant
- Teacher/teaching assistant
- Support worker
- Medical Professional (MD, RN, GP, etc)
- Medical Professional (Mental health practitioner)
- Other: [open text box]
- Prefer not to say

How many years of experience do you have working with autistic people?

- 0 - 4 years
- 5 - 10 years
- 10+ years
- Prefer not to say

Do you know someone who has gone through a language intervention?

- Yes
- No
- Unsure
- Prefer not to say

Do you deliver language interventions?

- Yes
- No
- Unsure
- Prefer not to say

What types of interventions do you deliver? [open text box]

**Researcher Demographics**

Researcher Demographics: This section has up to 5 questions.

What is your research role?

- Graduate student Masters level
- Graduate student PhD level
- Academic (early career)
- Academic (mid career)
- Academic (late career)
- Other (Please specify) [open text box]

How many years of experience do you have working in autism research?

- 0 - 4 years
- 5 - 10 years
- 10+ years
- Prefer not to say

Do you know someone who has gone through a language intervention?

- Yes
- No
- Unsure
- Prefer not to say

Do you conduct research on language interventions?

- Yes
- No
- Unsure
- Prefer not to say

What types of interventions do you research? [open text box]

**Language and Communication Questions**

Below are our definitions of communication and language. Please keep these in mind while taking this survey.

Communication is the process of successfully sharing ideas from one person to another. This can happen in many ways. Some examples are sounds, language, gestures, pictures, touch, smells.

Language is the use of words to express oneself. This can be spoken, written, or manual (signed). Language has rules that are known and shared by the people who use it.

Please answer the questions below to the best of your ability.

You can answer these questions however you want. This means you can write an answer, upload a picture you have drawn, or upload a voice note.

How important is **communication** to you? (0 = not important, 10 = very important) *Please slide the bar under the dial to indicate your rating*

Please tell us more about your answer. [open text box]

If you want to answer in a different way (like a picture or voice note), please upload your response here. You may only upload one file. The file uploaded can be up to 100MB.

How important is **language** to you? (0 = not important, 10 = very important) *Please slide the bar under the dial to indicate your rating*

Please tell us more about your answer. [open text box]

If you want to answer in a different way (like a picture or voice note), please upload your response here. *You may only upload one file. The file uploaded can be up to 100MB.*

Do you think that language is important for autistic people?

- Yes
- No
- Unsure

Please tell us more about your answer [open text box]

If you want to answer in a different way (like a picture or voice note), please upload your response here. *You may only upload one file. The file uploaded can be up to 100MB.*

**Language Intervention Questions & Video**

On the next page, we will ask you about language interventions for autistic people. Interventions are actions or treatments to try to improve something.

Below is a video about the research we did about language interventions. *If you require subtitles for the video, please download the plain text summary.*

We have told you about our research on language interventions. Now, we want to know your opinions.

What do you think are the benefits of language interventions for autistic people? [open text box]

If you want to answer in a different way (like a picture or voice note), please upload your response here. *You may only upload one file. The file uploaded can be up to 100MB.*

What do you think are the potential disadvantages/harms of language interventions for autistic people? [open text box]

If you want to answer in a different way (like a picture or voice note), please upload your response here. *You may only upload one file. The file uploaded can be up to 100MB.*
